# Practical Remarks on the Doses of Medicine

**Published:** 1878-06

**Authors:** R. H. Dalton

**Affiliations:** Los Angeles, Cal.


### PRACTICAL REMARKS ON THE DOSES OF MEDICINES.

BY R. H. DALTON, M. D., LOS ANGELES, CAL.

In prescribing medicines, physicians are usually governed by the doses laid down in the Dispensatory, and whenever an immediate impression in full force is required, we all know that the rule is right; the quantity specified is designed as an average and can not be required to meet the indications of every case, or every condition of the same case. Whenever it is desirable to thoroughly evacuate the bowels and stimulate the secretions at the same time, we do not hesitate to give fifteen to twenty grains of blue mass or calomel at once; but if our object is to cure an organic or functional disease of some standing, we would rather administer a few grains only, and keep up the impression by specific medication till its effects on the constitution are perceived. Even the ordinary alterative doses of such medicines will often fail by producing their effects too rapidly, Hence practitioners are sometimes unsuccessful by giving the right remedies in wrong doses. Mr. Abernethy, the pioneer of alterative medicine, was as much indebted for his great fame to skill in the manipulation of minute doses of medicine in chronic diseases as he was to surgical expertness; and he was probably the first practitioner who ever properly estimated the value of physiological science in surgery as a guiding light throughout its wide domain. To illustrate the principle I will adduce the following case which occurred in my practice four years ago:

A lady, Mrs. T., aged about fifty, had suffered for several months with myalgia, loss of muscular power, and more lately with anasarca. When first visited she was unable to stand alone or use her hands to feed herself, which seemed to be owing to the want of power to control the muscles rather than pain caused by their action. Finding the urine greatly diminished in quantity and containing albumen, I supposed it a case of Bright's disease of the kidneys, and hoping to relieve the patient by establishing the free action of the skin and bowels, I prescribed ten grains of Dover's powder at night, followed next day by thirty grains of co. powder of jalap every three or four hours till copious stools of a watery character were produced, but with no good effect whatever. After pursuing this treatment for four or five days, it was changed to an alterative and tonic course, and continued steadily for several days more but again with no benefit; in fact her condition grew worse all the time. Confirmed n the opinion that it was a case of Bright's disease, and having little hope of curing it, I decided to pursue a temporizing course, and ordered a large package of ten grain doses of the same powder (pulv. jalap co.) to be used simply as an alterative, directing a dose three times a day with no other medicine After an absence of twelve or fourteen days, I found her almost relieved of the dropsy, having some appetite, able to stand alone, and to feed herself without assistance. I learned that, after the second day of taking the powders, the bowels began to move gently two or three times a day with watery stools, while the kidneys acted much more freely. She continued the powders two weeks longer, sometime taking only two a day, when almost every sign of disease seemed to have disappeared, and her usual health and vigor soon returned. Now, in this case, the usual doses according to the Dispensatory, not only failed to cure the disease, but actually seemed to aggravate it, while very small doses certainly did remove every vestige of it.

Fifty years ago, as I well remember, the practice was to treat all acute diseases by violent perturbating remedies, such as copious blood letting, emetics, emeto-cathartics, drastic purgatives in the largest doses, etc., and at that time there was some excuse for such heroic measures. From some inscrutable telluric or meteorological cause, or from a peculiar constitutional diathesis of the whole human race, all acute diseases seemed to be much more intense than they have been since the prevalence of the cholera in 1832; and it is also remarkable that active depletion was then tolerated to a degree that would astonish a practitioner of the present day. The most reputable physicians bled *ad deliquium anima*, gave thirty or forty grains of calomel at a dose, and frequently premised their course of treatment, whatever it was to be, by a violent emetic; and if much pain was complained of, an extensive blister was promptly applied. The practice had been so lately in the hands of priests and barbers that it necessarily retained relics of their empiricism; and when we consider that physicial science was then in its infancy, and that pathological anatomy had scarcely been investigated, we may understand the reason of such powerful treatment. Even as late as 1827, an eminent Italian physician, Rasori, inculcated the doctrine of treating inflammatory diseases with large doses of tartar emetic, repeated every hour till tolerance was maintained, requiring sometimes thirty or forty grains in the course of a few hours. I myself have treated cases of rheumatism transferred to the brain, and pneumonia of high inflammatory grade, by that mode of practice, giving as much as thirty grains in three hours.

Heroic practice, though it may be deemed empirical and is carefully abjured by physicians of the present period, is nevertheless admissible in some cases of threatening importance, nor is it contrary to sound therapeutics; for we know by experience that if an organ or tissue has been recently changed from a normal to an abnormal condition by any pathological cause, we may expect it to be speedily restored by depletion, revulsion or some other mode of quick derivation. But if the lesion is one of long standing, with structural modifications, the same method of treatment will be apt to fail, as the conditions are very unlike. We should consider that chronic diseases are the usual sequelæ of imperfectly cured acute disorders, organic or functional, or the result of repeated hygienic violations, establishing conditions beyond the power of nature to ove1come. In this manner structural or interstitial changes have taken place, crippling the function of the organ and deranging by sympathy the general harmony of the whole economy. In such a case you may deplete, counter-irritate or resort to extreme revulsion, if you please, and when the effect ceases the lesion will still remain.

In medicine it is a truism that chronic diseases require chronic remedies. Hence the practitioner who may be skillful in the treatment of *acute* diseases may find himself at fault in those of a *chronic* form, if he fails to recognize the necessity of adapting means to ends. For instance, to attempt the cure of chronic indigestion, chronic constipation, or chronic neuralgia of the fifth pair of nerves, all of which may be usually traced to diminished peristaltic action of the bowels, by brisk cathartics to overcome the torpor, would be deemed malpractice; but to augment this motion to the degree of healthy action by the introduction of regular and persistent doses of medicine just sufficient to fulfill the object and no more, would be deemed good practice. And experience proves that the long continuance of such medication affords the best, if not the only, means of radically curing diseases of that character. If we could observe more closely the physiological actions of the system, we might gain a useful, practical lesson and learn to apply our art in imitation of a plan that admits of no failure. We would then recognize every function as the result of a stimulus or propelling force, whether visible or invisible; and while that force is normal in degree, that healthy action with all its agreeable concomitants must ensue; on the contrary, when abnormal, either in excess or deficiency, that abnormal or unhealthy action becomes inevitable. In fact, we would perceive that the human organization is, to some extent, like any other system of complex machinery, which when all its constitutent forces are sound and equal in all their bearings and running in grooves well supplied with lubricating material, can not be easily thrown out of gear; but if strained or propelled by irregular force, a screw be loosened or a joint weakned, every part of the machinery falls into disorder. To remedy this the skillful machinist, instead of counteracting irregular force by force, curbs the motion, tightens the screw or repairs the joint, and then the whole combination moves with usual regularity.

Let us turn our attention to a few illustrations to be found in the domain of Nature herself, and we may profit by the observation. The action of Carlsbad water in Germany exhibits wonderful power in the cure of many chronic diseases, and yet analysis proves that the whole quantity daily imbibed by an invalid contains but a few grains of the specific salts; and the same is true in regard to other celebrated mineral waters. The Marienbad water in Germany, used carefully for a length of time, is well known to be a successful remedy; but if taken too freely, it deranges the digestive system like poison. Again, the water used from the well at Wolverton and poisoning a number of people in 1848, was found to contain only one-sixth of the same salts held by that of Marienbad. The ex-royal family of France drank water from a well at Claremont, impregnated with only one grain of lead to the gallon, and all who drank it were poisoned. Even one-ninth grain of lead is capable of producing all the symptoms of poisoning. We need no more conclusive evidence than these examples afford, to prove the efficacy of very minute doses of medicine when taken regularly for a length of time; and if they really do possess such unquestionable power to impress the tissues and perturbate the functions of the system, why may they not be invoked to modify or supplant pathological conditions.

Being early impressed by such observations, when consulted in chronic disorders arising from retarded peristaltic action of the bowels, as I supposed, I frequently prescribed a solution of sulphate of soda, in doses every morning just sufficient to induce barely an operation after breakfast; and it was remarkable how little would suffice, provided a copious draught of water was taken with it.

It has been long known that the power of cathartics is augmented by combination, while the harshness of their action is diminished—a fact probably first announced by Dr. Paris in his Pharmacologia; and more recently it has been discovered that the addition of a very small portion of strychina to cathartics will accomplish the same result. Guided by these facts, I adopted the following formula many years ago for the relief of chronic constipation, which is a well known source of great annoyance to a great many people who go about seeming to be well, and is really the foundation of many serious chronic organic diseases:

℞ pulv. rhei, ʒj; aloes socot. ℈iv; scammon. ℈ij; saponis hispan. ʒj; strychniæ sulph. gr. iij; mel. despum. q. s. M. Ft. pil. No. 120. Sig: Take one pill every night.

After taking this pill for a night or two, the bowels begin to move gently every morning soon after breakfast, without the least griping or unusual disturbance, and they continue to act so as long as it is regularly taken. Only one operation being produced during the day. But when freshly made, a half pill will be sufficient to answer the purpose, and many persons never require more. I have taken one of these pills almost every night for the last thirty years, and expect to do so as long as I live. They are peculiarly adapted to nervous sick headache, to which sedentary persons are so liable, in fact, to all chronic disorders arising from tardy peristaltic motion of the bowels. The theory that a majority of such cases have their origin in this torpid condition of the *primæ viæ* is, doubtless, mainly correct; and if so, the pill is curative by performing the function of bile and pancreatic juice, which are the natural physiological forces to overcome it. The natural stimulus of the bowels (bile) being deficient, we adopt a harmless agent to supply its place as long as the habit of life which caused the deficiency continues; nor is there any reason to apprehend injury from the long continued use of the pill, for some of the healthiest persons I have known were in the habit of taking it almost every night for a quarter of a century without the slightest injury.

Before closing this paper, I will draw your attention to my mode of treating chronic emaciated cases of cholera infantum, by the steady administration of very minute doses of calomel, aided by stimulants and judicious regimen. As early as 1832 I adopted this mode of practice, and since that time I have treated an immense number of cases, occasionally departing from that plan to pursue some course recommended by high authority, but always with more or less disappointment, inducing me to return to my own method. The following formula was usually employed: ℞. calomel, gr. j; ipecac. grs. iv; pulv. zingib. grs. viij; sacch. alb. ʒss. Mix and divide into sixty-four powders; one to be given after each passage till the character is changed, then one only two or three times a day and night. In the meantime the little patient is thoroughly sponged all over twice a day with warm whisky or brandy; and if there is extreme emaciation, a quilted pad of pulv. cinchona, moistened with brandy, is worn constantly over the bowels. The food should be changed at least every second or third day, beginning with cream tea, made by adding one tablespoonful of sweet cream to three of boiling water and then sweetening; then boiled milk flavored with brandy; then arrowroot, sago, tapioca, farina, weak broth and the like, in a regular circle. Under this treatment cases will often thoroughly recover long before cold weather, as they scarcely ever do when managed in any other way.

